# Fertility education: what’s trending on Instagram

**DOI:** 10.1186/s40738-021-00095-6

**Published:** 2021-01-18

**Authors:** Alexandra Peyser, Liat Goldstein, Christine Mullin, Randi H. Goldman

**Affiliations:** 1 Division of Reproductive Endocrinology, Department of Obstetrics and Gynecology, Northwell Health, North Shore University Hospital, 300 Community Drive, Manhasset, New York 11030 USA; 2 Donald and Barbara Zucker School of Medicine at Hofstra/Northwell, Hempstead, NY USA

**Keywords:** Social media, IVF, Instagram, Infertility, Reproductive medicine

## Abstract

**Objective:**

To determine the prevalence, authorship, and types of fertility-related information shared on Instagram targeted toward a new patient interested in fertility options using hashtag and content analysis. Secondary outcomes included comparison of post content stratified by author type (physicians versus patients).

**Methods:**

A list of ten hashtags consisting of fertility terms for the new patient was derived. Content analysis was performed in April 2019 on the top 50 and most recent 50 posts for each hashtag to determine authorship and content type. The distribution of fertility terms in posts made by physicians was compared to that of patients and differences in use of terms were analyzed.

**Results:**

Our search yielded 3,393,636 posts. The two most popular hashtags were IVF (*N* = 912,049), and Infertility (*N* = 852,939). Authorship of the top posts for each hashtag (*N *= 1000) were as follows: patients (67 %), physicians (10 %), for-profit commercial groups (6.0 %), allied health professional (4.5 %), professional societies (1 %), and other (11 %). Of these posts, 60 % related to patient experiences, 10 % advertisements, 10 % outreach, and 8 % educational. Physicians were more likely to author posts related to oocyte cryopreservation compared to IVF, while patients were more likely to author posts about IVF (*p* < 0.0001).

**Conclusions:**

Over 3 million posts related to fertility were authored on Instagram. A majority of fertility posts are being mobilized by patients to publicly display and share their personal experiences. Concurrent with the rising utilization of planned oocyte cryopreservation, there is a trend toward physicians educating their patients about the process using social media as a platform. Physician participation on social media may offer a low-cost platform for networking and connecting with patients. Future studies examining the educational quality of posts by author type should be explored.

## Background

Social media has rapidly become an integral part of our daily routines. Communication via user-generated content enables interaction and collaboration among healthcare communities and patients [1]. Patients have the opportunity to utilize social media to inquire information about their symptoms, diagnosis, and treatment. The vast majority of Americans report using the internet to inquire about health-related questions [2, 3].

Instagram is a free online social networking service that is currently used by 67 % of American young adults aged 18–29 years, and 47 % of adults aged 30–49 [1]. The platform enables users to post pictures and videos to their profiles, add a caption, and use hashtags (# symbol) to describe photos and categorize posts into searchable topics that other users can find. Users can follow specific hashtags and any number of accounts to view a steady stream of personalized content. Users can also search by hashtag to view desired content posted in aggregate and “share”, “like”, or “comment” on posts [4]. A recent poll showed that the average user spends 21.2 minutes on Instagram each day, with the 18–29 age group spending the most time at 30 minutes [5].

Online availability of health-related information has increased significantly in recent years [6]. Social media has changed the way people seek and share information on healthcare despite the potential lack of information quality control. However, little information is available on the use, risks, and benefits of using social media to educate the general public, patients, and healthcare professionals [7].

Increasing numbers of patients are now sharing their (in)fertility journeys through Instagram [7, 8]. Using hashtags, patients can share their experiences or find others going through similar circumstances. Physicians are able to utilize Instagram as a platform for sharing evidence-based medical education to inform the public on any health care subject [5, 9–11]. Despite the rising influence of this particular platform, the literature remains scarce regarding the use of Instagram within the field of reproductive medicine and infertility. There are few peer-reviewed studies that quantitatively evaluate fertility-related content on Instagram, and more importantly, there are limited published reports indicating who is responsible for these posts [12].

A recent article by Blakemore et al. analyzed individual accounts from “Fertility Influencers” on both Twitter and Instagram to determine the topography of fertility-related information on social media. They found that the majority of influential accounts were from patients, with a paucity of board-certified Reproductive Endocrinologist and Infertility specialists contributing to this space. Authors analyzed the 5 most recent posts by fertility influencers (39 on Twitter and 28 on Instagram) and reported the top 11 hashtags most used by those influencers. While that study focused on fertility influencers and their most commonly used hashtags, this study was designed to determine what a first time patient desiring fertility treatment may find as they begin their search on Instagram.

The purposes of this study are therefore: (1) to determine the prevalence, authorship, and types of fertility-related information targeted toward new fertility patients on Instagram and (2) to compare the content of posts authored by physicians versus patients.

Methods.

**Fig. 1:**
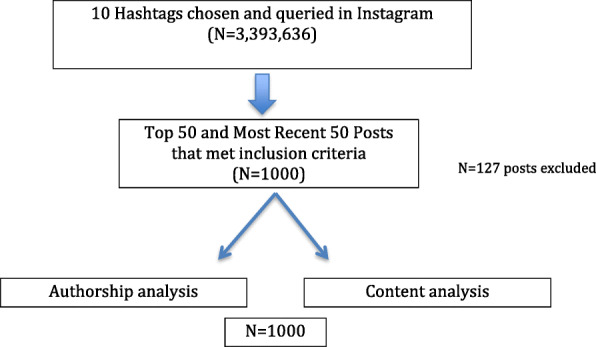
Flow diagram depicting experimental design

A list of ten hashtags consisting of fertility terms were derived which included: IVF, Infertility, Fertility, IVF Journey, Infertility Awareness, IVF Support, Fertility Awareness, IVF Success, Egg Freezing and Egg Retrieval. These terms were chosen based on what a first time IVF or planned oocyte cryopreservation patient may search for as they gather information regarding fertility options. Six of the chosen hashtags were considered some of the most common posted by fertility social media influencers [12]. Our methods using Instagram hashtag search and analysis were adapted from previous studies in other fields of medicine (Park et al. and Qin et al.) [13, 14]. The total number of posts using each hashtag was recorded via an automatic count that is generated by Instagram. Content analyses were performed by two of the study investigators (AP and LG) to qualitatively evaluate each of the top 50 and most recent 50 posts for each hashtag. Clear definitions for each category were designed to minimize any potential bias. A total of 100 posts were analyzed for each hashtag, for a total of 1000 posts reviewed (Fig. 1). Authorship and content type for each hashtag were reviewed on April 17, 2019. Top posts were determined by Instagram via a proprietary algorithm consisting of parameters including the number of likes, comments, and user engagement on the posts.

Content type was divided into the following categories: educational, patient experience, outreach, advertisement, research, personal (unrelated to a diagnosis), and other. Educational posts included posts where the primary goal was to provide education on fertility issues (e.g., a physician describing the basic fertility work up). Outreach posts involved posts where the author was trying to engage the user (e.g., “comment below if you have heard of anti-müllerian hormone!”). Advertisements included a clear promotional goal (e.g., an office offering a free initial fertility consult).

Authorship categories included: patient, physician, allied health professional (e.g., nurse, psychologist, doula), professional society, for-profit commercial group, and other (law firms, community organizations/non-profit groups, exercise professionals or other professionals not meeting criteria as allied health professionals). This study was reviewed and found to be exempt by the Northwell Health Institutional Review Board.

We excluded any posts that were not in English, were “reposts” of another Instagram account, or included videos. Videos were excluded to strictly capture differences among posts of photos with captions. All included posts were then designated with their respective categories for content type and authorship. The distribution of fertility terms in posts made by physicians was compared to that of patients, and differences in the use of specific hashtags were analyzed using chi-square analyses; *p* < 0.05 determined statistical significance.

## Results

Our search yielded 3,393,636 posts. Table 1 demonstrates the number of posts for each hashtag. The 5 most popular hashtags were IVF (*N* = 912,049), Infertility (*N* = 852,939), Fertility (*N* = 535,614), IVF Journey (*N* = 431,839) and IVF Success (*N* = 285,633).

**Table 1:** Fertility-related hashtags and number of posts on instagram in April, 2019

Hashtag	Total posts (N)	Percentage (%)
IVF	912,049	26.9
Infertility	852,937	25.1
Fertility	535,614	15.8
IVF journey	431,839	12.7
IVF success	285,633	8.4
Infertility awareness	189,507	5.6
IVF support	111,669	3.3
Fertility awareness	46,536	1.4
Egg retrieval	14,644	0.4
Egg freezing	13,208	0.4
Total	3,393,636	

Egg Freezing and Egg Retrieval had the lowest number of posts (*N* = 13,208 and *N* = 14,644, respectively).

Authorship of the top 50 and most recent 50 posts for each hashtag (*N* = 1000) were as follows: patients (67 %), physicians (10 %), for-profit commercial groups (6.0 %), allied health professional (4.5 %), professional societies (1 %), and other (11 %). Of these posts, 60 % related to patient experiences, 10 % advertisements, 10 % outreach, 8 % educational, and 6 % personal posts unrelated to diagnoses (Table 2). When comparing authorship of posts by hashtag, seven of the ten were posted more frequently by patients than physicians (Table 3). Physicians were more likely to author posts related to oocyte cryopreservation compared to IVF, while patients were more likely to author posts about IVF (*p *< 0.0001).

**Table 2:** Content and authorship of each instagram hashtag

Content:	Hashtag								**IVF success (N)**	**IVF support (N)**	**Percentage of total posts (%)**
	**Egg freezing (N)**	**Egg retrieval (N)**	**Fertility (N)**	**Fertility awareness (N)**	**Infertility (N)**	**Infertility awareness (N)**	**IVF (N)**	**IVF journey (N)**			
Educational	20	-	8	31	3	5	6	4	2	1	**8.0**
Personal posts unrelated to their diagnoses	6	4	8	1	9	6	9	4	9	2	**5.8**
Patient experiences pertaining to their diagnoses	34	89	37	38	59	63	60	80	55	83	**59.8**
Outreach posts	20	2	19	6	15	6	12	2	12	5	**9.9**
Advertisements	12	4	20	16	6	15	4	10	11	7	**10.5**
Research	-	-	-	-	-	-	-	-	-	-	**0**
Other	8	1	8	8	8	5	9	-	11	2	**6.0**
Total:	100	100	100	100	100	100	100	100	100	100	
Author:											
Allied Health Professional	6	-	4	19	2	2	-	5	2	5	**4.5**
Patients	37	91	47	46	73	74	75	82	65	84	**67.4**
Physicians	34	5	11	12	1	4	6	4	17	2	**9.6**
Professional society	2	-	1	-	1	-	-	-	7	-	**1.1**
For profit commercial groups	5	3	11	17	7	5	5	4	-	3	**6.0**
Other	16	1	26	6	16	15	14	5	9	6	**11.4**
Total:	100	100	100	100	100	100	100	100	100	100	

**Table 3:** Frequency of posts among physicians versus patients

Hashtag	Physicians N (%)	Patients N (%)	*P* value
Egg freezing	34 (35)	37 (6)	**< 0.0001**
Egg retrieval	5 (5)	91 (15)	**0.012**
Fertility	11 (11)	47 (8)	0.183
Fertility awareness	12 (13)	46 (7)	0.083
Infertility	1 (1)	73 (12)	**0.001**
Infertility awareness	4 (4)	74 (12)	**0.024**
IVF	6 (6)	75 (12)	0.096
IVF journey	4 (4)	82 (13)	**0.011**
IVF sucesss	17 (18)	65 (10)	**0.034**
IVF support	2 (2)	84 (13)	**0.001**
Total	96	626	

Bold indicates significance

Posts with the hashtag ‘Infertility Awareness’ were authored more by patients compared to physicians (74 % vs. 4 % *p* = 0.024), with the majority of patients utilizing hashtags to describe their diagnoses. ‘IVF Journey’ and ‘IVF Support’ were both authored significantly more by patients compared to physicians (82 % vs. 4 %, *p* = 0.011 and 84 % vs. 2 %, *p* = 0.001, respectively). Educational posts were largely related to ‘Fertility Awareness’ (31/80, 38 %) and ‘Egg Freezing’ (20/80, 25 %). When patients posted about their diagnoses, the hashtags ‘Egg Retrieval’ (89/598, 15 %) and ‘IVF Support’ (83/598, 14 %) were used the most. Advertisements utilized ‘Fertility’ in 19 % (20/105) of posts and ‘Egg Freezing’ in only 11 % (12/105). While 15 % of total posts were authored by physicians, allied health professionals and professional societies, there were zero posts related to research and only 8 % related to education.

## Discussion

Our study demonstrated an abundance of information related to fertility on Instagram with 66 % of posts directed toward patient experience and authored by patients. Only 10 % of posts were authored by individual physicians. Ten percent of posts were advertisements directed at reproductive-aged women.

The types and content of posts authored by patients and physicians significantly differed. Patients were more likely to post about their IVF experience, particularly sharing their infertility journeys, including both success and failures with treatment. In contrast, physicians were more likely to post about planned oocyte cryopreservation. This may be due to physicians’ desire to educate the public about planned oocyte cryopreservation and promote informed reproductive decision-making. In addition, the employer-based expansion of insurance benefits to include planned oocyte cryopreservation and the increase in number of women delaying childbearing may contribute to the increased commentary by physicians [15]. Patient inquiries regarding the cryopreservation process have increased, whereas IVF has been availed by patients and couples since the 1970s [16]. Competition between centers in geographically condensed areas as well as the rise in direct-to-consumer companies that promote fertility testing may additionally lead to increased incidence of these posts [17].

Hashtags allow patients to join online communities and enable conversations about desired health care topics outside of the clinical setting (office/hospital) and from any computer or smart device. For patients, it may be empowering to find a sense of community and have the chance to communicate with others who are going through similar processes [18–20]. Results of a recent study demonstrated that a majority of patients post about their miscarriage experience on Instagram to seek support and community from others. Some patients seek medical guidance after witnessing others’ experiences with treatment. Other studies have suggested that online social groups have allowed for the normalization of patient experience and reduced social isolation, therefore improving patients’ psychosocial mindset regarding infertility [19, 20]. In 2018, Omurtag et al. studied patient preference for social media utilization and content at three infertility clinics in the United States. They found that infertility patients desire the use of social media for both educational content as well as a resource for dealing with the emotional consequences of infertility. Eighty percent of survey respondents felt that social media improved their overall patient experience in the infertility clinic. The majority (60 %) of survey respondents used Instagram [21].

Despite the recognized role of social media in providing health-related information, many medical providers and public healthcare institutions are reluctant to use social media platforms. Providers need to be wary of maintaining patient confidentiality online and abiding by HIPAA. Providers must also be aware of workplace policies surrounding use of social media, the time commitment of posting content, and the implications of engaging in discussions online. Many medical societies have published guidelines on the appropriate use of social media. The American College of Obstetricians and Gynecologists (ACOG) was one of the first to offer guidance to physicians [22]. Their recently revised committee opinion in 2019 states that social media is “not only acceptable for the modern practicing physician, but has become a necessary element for relating to patients and practicing medicine [23].” The American Medical Association (AMA) describes six considerations for ethical use of social media, encouraging protection of patient privacy, realizing permanence, alerting colleagues to suspected inappropriate use, maintaining patient boundaries, realizing that actions online may negatively affect their reputation amongst patients and colleagues, and considering creating separate personal and professional accounts [24].

Collaboration of physicians within the social media community is motivated by the passion to spread knowledge. A recent review published on the online portal FertStert Dialog analyzed how physicians in reproductive medicine are using social media to influence the fertility community [25]. Physicians expressed a desire to connect with patients and explain infertility and other aspects of women’s health. They acknowledged that the millennial generation, who are now utilizing fertility services, will undoubtedly employ social media as an adjunct to clinic websites. Providers find it rewarding to connect with the fertility community at large and have found that posts containing educational content are of highest interest [26]. In addition, some physicians may post about their own personal experiences with diagnostic tools, procedures, medications, and supplements. Highly-followed providers may be considered “influencers” and be compensated by companies for their posts.

The presence of physicians on social media provides an opportunity to help facilitate evidence-based information-sharing and debunk factually-incorrect myths and misconceptions. However, our results have demonstrated that physician presence is lacking with only 10 % of posts authored by physicians and 1 % by professional societies. In addition, as only a small percentage of posts were considered educational and none involved research, there is a strong need for physicians to enter the Instagram space to provide factual information to the public as well as to describe innovative and ongoing research in our field. Future initiatives to enhance physician involvement while ensuring patient privacy on social media should be encouraged.

Limitations to our study include the use of a single social media platform with results that may not be generalizable to other platforms. This study was based on an evaluation of posts during a limited time frame, while Instagram data is continuously changing. The chosen hashtags may not be inclusive of all possible hashtags a new patient may search for. For example, the hashtag ‘ttc’ (trying to conceive) is more popular when one performs an Instagram search. However, we believe it is less likely to be a term one uses upon their first search for more information, particularly about oocyte cryopreservation. Finding the most common fertility hashtags may in fact be a futile endeavor due to the fluidity of social media. In addition, some accounts were ‘private’ and therefore inaccessible, not allowing us to view every possible post. The exclusion of videos from our study could have biased our results if physicians/educational groups posted a higher percentage of videos. Importantly, content on social media may lack accuracy as there is no review process to ensure that posts are evidence-based.

Strengths of our study include the large number of posts analyzed and that it is one of the few studies evaluating the fertility social media webspace. We have identified that patients are interested in describing their experiences with IVF and that there is a relative paucity of posts authored by physicians. A benefit to this study is that it introduces social media to providers who may not be familiar with this form of communication.

Further research is needed to examine the educational quality of posts by author type, explore the impact of social media on the patient-physician relationship, and evaluate how social media influences medical decision making. Expanding the analysis to include a larger number of posts using machine-based learning may allow for a more nuanced understanding of social media usage by patients and providers over a longer time frame.

## Conclusions

Social media provides a powerful tool to inform, engage, and communicate with the fertility community. It will exponentially grow larger as the millennial and Gen Z generations begin to reach their reproductive prime. Physicians have an opportunity to connect with and educate a large number of patients looking for support by authoring evidence-based posts on social media.

## Data Availability

The datasets used analyzed during the current study are available from the corresponding author on reasonable request.

## Abbreviations

IVF: In Vitro Fertilization
AMA: American Medical Association

## References

[1] Share of U.S. adults using social media, including facebook, is mostly unchanged since 2018 [Internet]. Pew Research Center. 2020 [cited 28 July 2020]. Available from: https://www.pewresearch.org/fact-tank/2019/04/10/share-of-u-s-adults-using-social-media-including-facebook-is-mostly-unchanged-since-2018/.

[2] Diaz JA, Griffith RA, Ng JJ, Reinert SE, Friedmann PD, Moulton AW (2002). Patients’ use of the Internet for medical information. J Gen Intern Med.

[3] Benabio J (2013). The value of social media for dermatologists. Cutis.

[4] “Instagram help center.” How do I use hashtags on instagram? | Instagram Help Center, https://help.instagram.com/351460621611097.

[5] Moorhead SA, Hazlett DE, Harrison L, Carroll JK, Irwin A, Hoving C (2013). A new dimension of health care: systematic review of the uses, benefits, and limitations of social media for health communication. J Med Internet Res..

[6] Tonsaker T, Bartlett G, Trpkov C (2014). Health information on the Internet: gold mine or minefield?. Can Fam Physician.

[7] Hirsch M, Aggarwal S, Barker C, Davis CJ, Duffy JMN (2017). Googling endometriosis: a systematic review of information available on the Internet. Am J Obstet Gynecol.

[8] Kedzior SGE, Bianco-Miotto T, Breen J (2019). It takes a community to conceive: an analysis of the scope, nature and accuracy of online sources of health information for couples trying to conceive. Reprod Biomed Soc Online.

[9] Abraham LB, Morn MP, Vollman A. Women on the web: how women are shaping the internet. http://www.comscore.com/Press_Events/Presentations_Whitepapers/2010/Women_on_the_Web_How_Women_are_Shaping_the_Internet. Accessed 12 Jan 2020.

[10] Chou WY, Hunt YM, Beckjord EB, Moser RP, Hesse BW (2009). Social media use in the United States: implications for health communication. J Med Internet Res.

[11] Liu 11ChouWY, Post B, Hesse S (2011). Health-related internet use among cancer survivors: data from the Health Information National Trends Survey, 2003–2008. J Cancer Surviv.

[12] Blakemore JK, Bayer AH, Smith MB (2020). Infertility influencers: an analysis of information and influence in the fertility webspace. J Assist Reprod Genet.

[13] Park JH, Christman MP, Linos E (2018). Dermatology on Instagram: an analysis of hashtags. J Drugs Dermatol.

[14] Qin LA, El-Neemany D, Winkler H, Shalom D (2020). #Urogyn: what’s trending on instagram? A cross-sectional observational study. Female Pelvic Med Reconstr Surg.

[15] Argyle CE, Harper JC, Davies MC (2016). Oocyte cryopreservation: where are we now?. Hum Reprod Update.

[16] Gunnala V, Schattman G (2017). Oocyte vitrification for elective fertility preservation: the past, present, and future. Curr Opin Obstet Gynecol.

[17] Kyweluk MA (2020). Quantifying fertility? Direct-to-consumer ovarian reserve testing and the new (In)fertility pipeline. Soc Sci Med.

[18] Mercier RJ, Senter K, Webster R (2020). Henderson riley A. Instagram users’ experiences of miscarriage. Obstet Gynecol.

[19] Kingod N, Cleal B, Wahlberg A, Husted GR (2017). Online peer-to-peer communities in the daily lives of people with chronic illness: a qualitative systematic review. Qual Health Res..

[20] Nambisan P (2011). Information seeking and social support in online health communities: impact on patients’ perceived empathy. J Am Med Inform Assoc..

[21] Broughton DE, Schelble A, Cipolla K, Cho M, Franasiak J, Omurtag KR (2018). Social media in the REI clinic: what do patients want?. J Assist Reprod Genet.

[22] Professional Use of Digital and Social Media (2015). ACOG committee opinion, number 622. Obstet Gynecol.

[23] Professional Use of Digital and Social Media (2019). ACOG committee opinion, number 791. Obstet Gynecol.

[24] American Medical Association. Opinion 9.124 professionalism in the use of social media. Code of Medical Ethics. https://www.ama-assn.org/delivering-care/ethics/professionalism-use-social-media Accessed 11 Jun 2020.

[25] Broughton D, Chen S, Crawford N, Feinberg E, Forman E, Grindler N, Kallen A, Kudesia R, Perfetto C, Shahine L, Trolice M, Omurtag K. 2020. Fertility social media influencers, part 1: your brand and your narrative. Fertility and Sterility Dialog. Available at: https://www.fertstertdialog.com/users/16110-fertility-and-sterility/posts/52945-omurtag-consider-this-part-1.Accessed 22 Jan 2020.

[26] Broughton D, Chen S, Crawford N, Feinberg E, Forman E, Grindler N, Kallen A, Kudesia R, Perfetto C, Shahine L, Trolice M, Omurtag K. 2020. Rise of the social media influencer in fertility care, part 2: doin’ it (professionally) for the “Gram”. Fertility and Sterility Dialog. Available at: https://www.fertstertdialog.com/users/16110-fertility-and-sterility/posts/54156-omurtag-consider-this-part-2. Accessed 22 Jan 2020.

